# Undifferentiated embryonal sarcoma of the liver treated with associating liver partition and portal vein ligation for staged hepatectomy in a young adult: A case report

**DOI:** 10.1016/j.ijscr.2019.11.052

**Published:** 2019-11-30

**Authors:** Dmitri Schepelew, Tim Reese, Katja Horling, Christian Frenzel, Karl J. Oldhafer

**Affiliations:** a Department of General and Visceral Surgery, Centre for Oncological Surgery, Asklepios Klinik Barmbek, Hamburg, Germany; b Semmelweis University Budapest, Faculty of Medicine, Asklepios Campus Hamburg, Germany; c Institute for Hematopathology, Hamburg, Germany; d II. Medical Clinic and Polyclinic, Centre for Oncology, University Medical Centre Hamburg-Eppendorf (UKE), Germany

**Keywords:** Surgery, Liver resection, Undifferentiated embryonal sarcoma of the liver, Associating liver partition and portal vein ligation for staged hepatectomy

## Abstract

**Introduction:**

Embryonal sarcomas of the liver (ESL) are extremely rare solid tumors appearing mainly in children. The therapeutic standard for an ESL is a margin free resection combined with chemotherapy. The Associating Liver Partition and Portal Vein Ligation for Staged Hepatectomy (ALPPS) procedure as a surgical therapy offers a curative approach for liver tumors of various origins where the future liver remnant (FLR) would be insufficient after a one-staged (extended) hemihepatectomy.

**Presentation of Case:**

A 19-year-old patient was diagnosed with an undifferentiated embryonal sarcoma of the liver (UESL) in the right liver lobe with oligometastatic spread to the lungs.

After neoadjuvant chemotherapy remission was enough to plan a resection of the liver tumor. During the operation we changed our strategy from one-stage hepatectomy to ALPPS because of borderline FLR and macroscopic and histologic liver damage to avoid posthepatectomy liver failure. The interstage and postoperative course of the patient was uneventful beside postoperative bile leakage, which was treated by interventional drainage and stenting.

**Discussion:**

The ALPPS-procedure as a comparatively new surgery was considered over a portal vein ligation or embolization. ALPPS shows a faster hypertrophy compared to standard one-staged hemihepatectomy with decreased or similar proliferation, apoptosis or angiogenesis (at least for CRLM)

**Conclusion:**

In experienced centers the ALPPS-procedure is evolving as the safer approach in hemihepatectomys where the FLR is critical. Additionally, ALPPS can serve as an intraoperative option when liver volume and quality seem not to be sufficient and is to be considered when facing new tumor-entities.

## Highlights

• First Case Report about ALPPS for the treatment of an undifferentiated embryonal sarcoma of the Liver.
• The feasibility of the ALPPS procedure in a newly faced tumor-entity.
• Importance of an intraoperative histological evaluation of the parenchyma to estimate the individual risk for liver failure.

## 1 Introduction

Embryonal sarcomas of the liver (ESL) are extremely rare in children, with an incidence of 1 in 1.000.000, even less in adults. Most common types of soft tissue sarcomas are rhabdomyosarcoma (57 %), neuroectodermal (10 %) and synovial tumors (8 %). Of primary liver tumors in children, the hepatoblastoma and the hepatocellular carcinoma are the most common, followed by the focal nodular hyperplasia and then the embryonal sarcoma. The undifferentiated embryonal sarcoma of the liver (UESL), firstly identified as an independent clinicopathologic type of sarcoma in 1978, is seen in about 2 % [[1], [2], [3]]. Typically, there is no pre-existing liver disease. Less than 70 cases of UESL were reported in adults till 2008 [4]. Definitive diagnosis and the concept of treatment can only be established after histological confirmation. The only documented curative treatment is margin-free radical surgical resection, conjoined with neoadjuvant therapy if oncologically necessary [5].

The Associating Liver Partition and Portal Vein Ligation for Staged Hepatectomy (ALPPS) is a two-stage hepatectomy with an extensive regenerative response. The procedure provides the possibility of resecting a liver tumor leaving an otherwise insufficient future liver remnant (FLR) for an one-staged operation by inducing liver hypertrophy via a two-step approach [[6], [7], [8]].

Purpose of this case report is to show the efficacy of ALPPS as an established method to increase the resectability rate and the important role of intraoperative histological analysis. To our knowledge, we describe the first UESL treated with ALPPS.

This paper has been reported in line with the SCARE criteria [9].

## 2 Case report

**Fig. 1:**
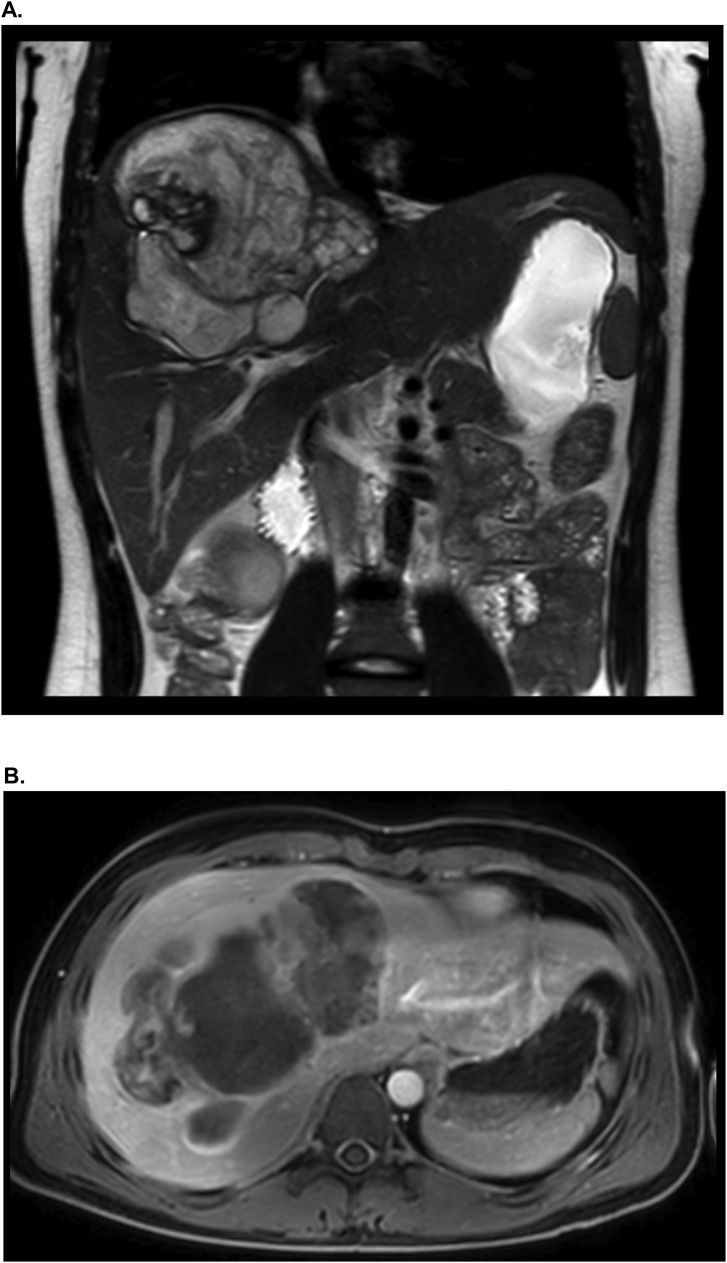
Preoperative MRI shows a heterogenic tumor mass in the right liver lobe before chemotherapy. The lesion invades segment IVa and V-VIII. A: Coronal MRI B: Axial MRI.

A 19-year-old male patient presented with intermittent shoulder pain and a palpable mass in the right upper quadrant, causing abdominal discomfort. MRI-imaging revealed a hyper intense inhomogeneous mass located in the right lobe of the liver with an approximate size of 9,5 cm in diameter and five nodules in both lower lobes of the lung (Fig. 1). The working diagnoses was a hepatic cyst/alveolar echinococcosis.

Definitive diagnosis was made by biopsy of the pulmonary nodule of the left lower lobe, showing an undifferentiated pleomorphic embryonal sarcoma. The staging was cT2b cN0 cM1 (PUL), UICC IV, Intergroup Rhabdomyosarcoma Studies (IRS) stage 4. The multidisciplinary conference recommended neoadjuvant chemotherapy followed by a radical surgical resection.

**Fig. 2:**
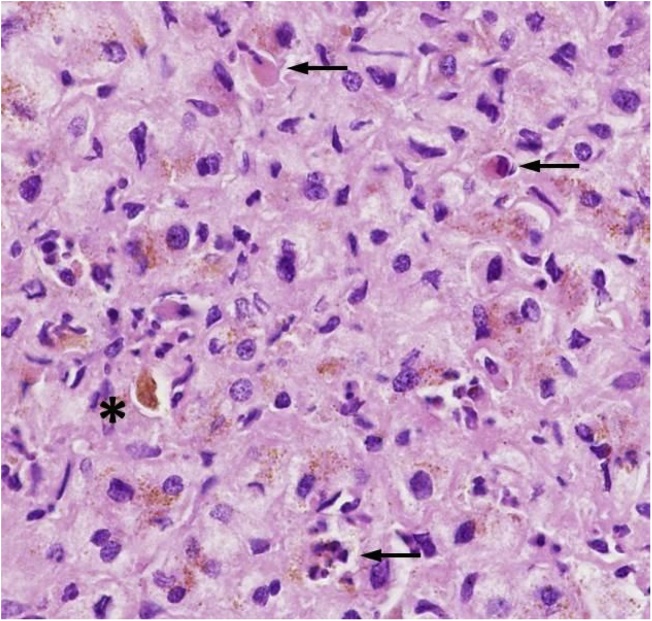
Histology of the liver parenchyma shows mild cholestasis (asterisk) and spotty necrosis (arrow).

**Fig. 3:**
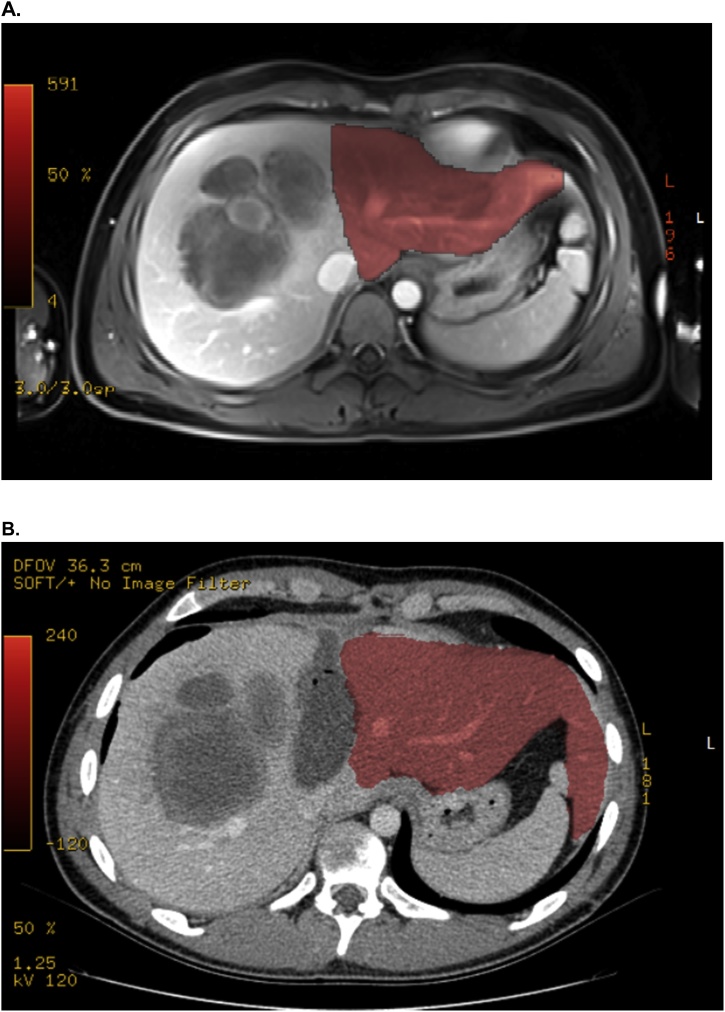
A: Volumetry before step I with 28,4 % sFLR (red section); B: Volumetry before Step II showing 57,3 % sFLR.

Neoadjuvant chemotherapy was performed according to the Cooperative Soft Tissue Sarcoma Study Group (CWS-2014 guidance, treatment guidelines for soft tissue sarcoma and soft tissue tumors) for 13 weeks, with four cycles of CEVAIE. Primary tumor and pulmonary metastasis showed a good response and shrinking in restaging. The patient had normal liver laboratory values and a preoperative LiMAx of 479 μg/kg/h. A hemihepatectomy (right lobe + segment 4b) with a sFLR of 28,4 % of segments I-IVa was planned (Fig. 3A). Intraoperatively, the liver parenchyma was firm and macroscopically a chemotherapy-associated liver damage was suspected. Therefore, we went for an intraoperative histopathological analysis of the parenchyma in fresh-frozen sections. This revealed cholestasis, beginning portal fibrosis and single cell necrosis (Fig. 2). Additionally, invasion of not only the right, but also the middle hepatic vein was noted. Consequently, a FLR of 28 % combined with injured liver parenchyma seemed not to be adequate for a one-stage liver resection. To reduce the risk of a posthepatectomy liver failure, a hypertrophy concept was necessary. We decided to proceed with ALPPS for the induction of hypertrophy. A classical step I with a complete transection between segments I-IVa and IVb-VIII was done down to the vena cava. Intraoperative blood loss was 1.200 ml. No complications occurred and on POD 5 the patient was discharged with normal liver function.

**Fig. 4:**
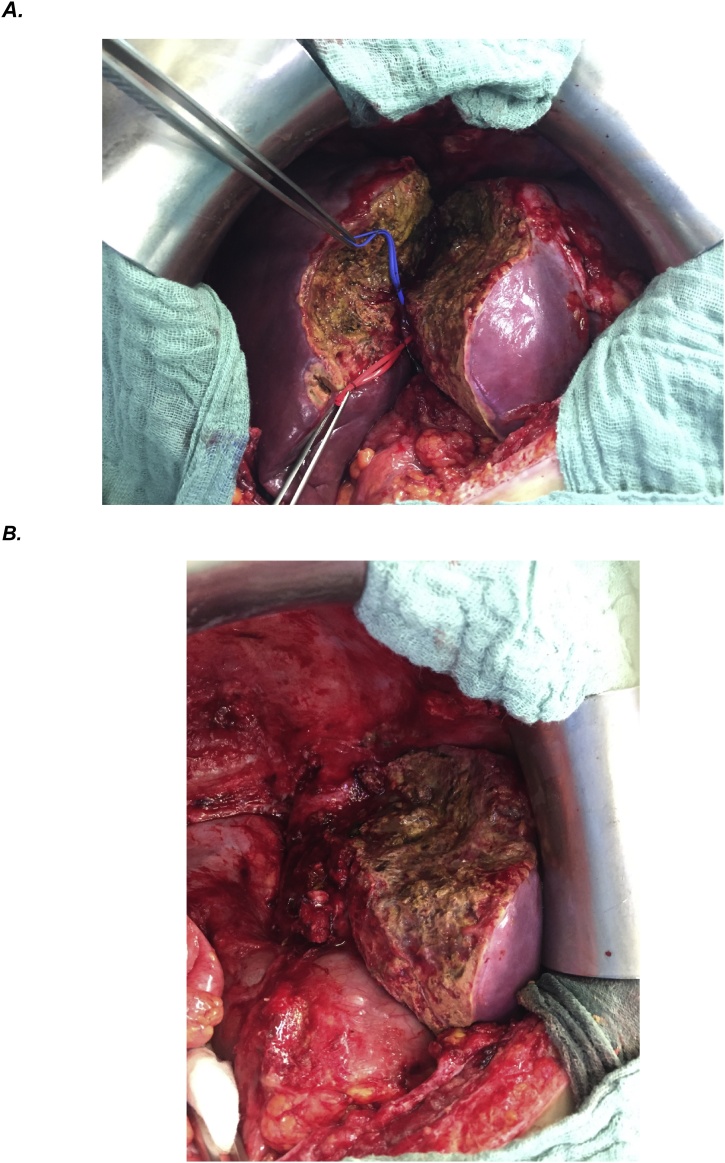
A: Intraoperative situs during step II showing the parenchymal splitting line and left-lobe-hypertrophy induced by step I. The right bile duct is tagged by a blue and the right hepatic artery by a red band. B: Situs after complete resection of the right liver lobe.

**Fig. 5:**
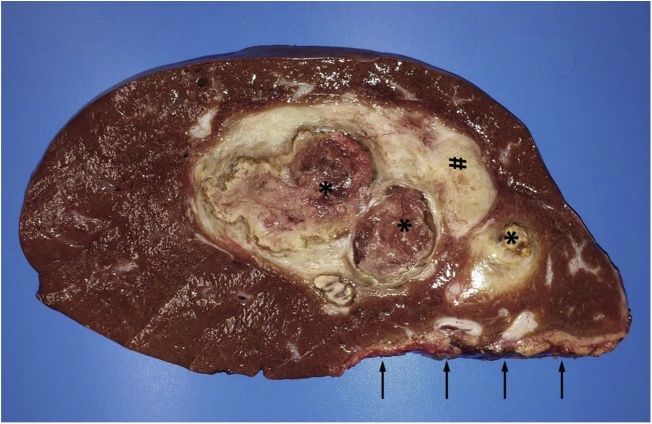
Liver specimen after resection showing tumor free resection sites margin (arrow) with a minimum distance of 0.5 cm. The tumor area presented large necrosis and haemorrhage (asterisk) as well as a fibrous pseudocapsule (hashmark).

Nine days after step I, re-volumetry based on a CT scan revealed a sFLR of 57,3 % (Fig. 3B). The following day, step-II was performed and the tumor-bearing liver part removed. Fig. 4 shows the intraoperative situs before and after resection. The specimen is shown in Fig. 5. LiMAx test on POD 1 was 269 μg/kg/h, presenting a sufficient liver function of the remnant.

**Fig. 6:**
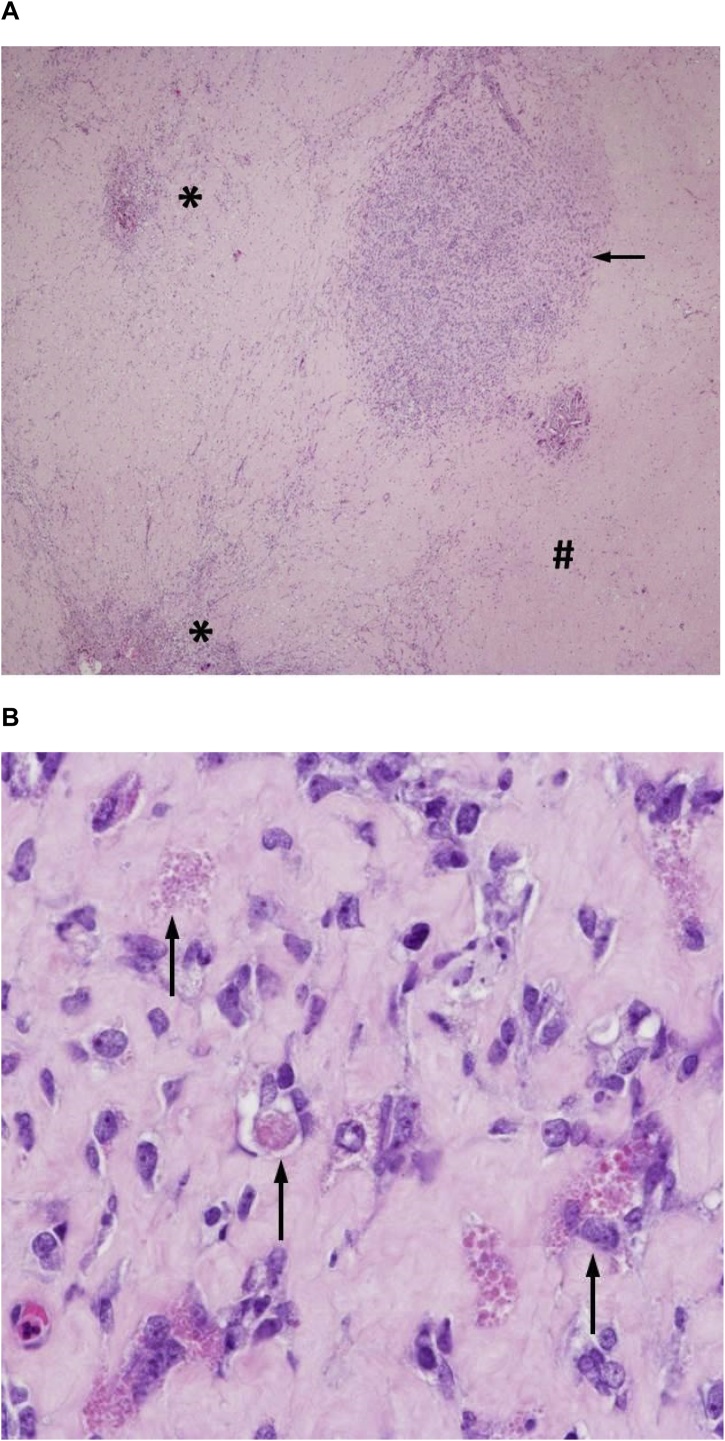
In sclerotic areas (A, hashmark) with resorptive changes (A, asterisk) only a few small spots of residual vital tumor cells (A, arrow) with the characteristic hyaline globules are seen (B, arrow).

The patient developed bile leakage, which was treated with interventional drainage and ERC. Otherwise, the postoperative course was uneventful and the patient was discharged in good conditions on POD 14. The pathological work up demonstrates tumor free margin. Extensive sampling of the tumor bed shows broad areas of fibrosis and necrosis with haemorrhage and resorptive changes with infiltrates of foamy histiocytes. Only a few small spots of residual vital sarcoma could be detected (Fig. 5, Fig. 6). The therapy-induced tumor regression grade was 99 %. After recovery from surgery, chemotherapy according to the CEVAIE protocol (CWS-2014 guidance) will be resumed and a complete resection of the oligometastatic pulmonary disease is planned.

## 3 Discussion

Embryonal sarcoma is an exceptionally rare diagnosis with almost no improvement in 5-year-survival-rate over the past 40 years [10]. The higher the age at diagnosis, the lower is the 5-year-survival rate. Among the age-group of 15–29 years the 5-year survival is about 45 %. Margin-free resection is the only known chance of cure. A study of 30 patients with primary hepatic sarcoma showed a 5-year disease specific survival of 64 % if R0 resected, otherwise no 3-year survivor was documented [5].

Developed in the last decade, ALPPS offers a surgical option for patients with extensive liver lesions, bilobular liver tumors (metastasis invading or tumor masses expanding over both lobes) or a low FLR. After transection of the liver and ligation of the portal vein of the tumor-bearing liver lobe, a rapid hypertrophy of the FLR is induced [8]. Therefore, the risk of posthepatectomy liver failure is reduced. The full spectrum of treatable liver lesions is evolving still [2,[11], [12], [13]]. However, an initial throwback of this procedure was the initial high rate of major complications, which could be reduced with a careful patient selection and avoiding possible risk factors [[14], [15], [16]].

In this case report, we present the first case of UESL treated with ALPPS. The rarity of this highly malignant disease is also based on its rapid tumor growth and early metastasis. Definitive and early diagnosis is impeded by its nonspecific symptoms, radiographic similarity to hepatic cysts and alveolar echinococcosis linked with the only available validation being histological investigation. Neoadjuvant chemotherapy to ensure possibility for oncological operation will therefore prevail, as margin-free resection is the only known real cure. With a tumor invading the right liver lobe and leaving a sFLR of 28,4 %, the spectrum of possibilities is limited. With the intraoperatively found invasion of the right and middle hepatic vein and because of a firm liver and intraoperative histological evaluation of the parenchyma we did not perform the initially planned one-staged right hemihepatectomy and changed the strategy. To reduce the risk of posthepatectomy liver failure, a complete transection of the liver and ligation of the right portal vein was performed. The aim was a rapid hypertrophy and timely completion of the resection. The risk for complications or mortality in this patient was suspected as very low. For our center it was also the first patient treated with ALPPS at this young age. A FLR growth of approximately 100 % in 9 days strengthened our concept during the course. Other options, such as portal vein embolization or ligation could have failed because of slower hypertrophy and the risk of tumor progression before liver resection. Besides, ALPPS shows a faster hypertrophy compared to standard one-staged hemihepatectomy with decreased or similar proliferation, apoptosis or angiogenesis (at least for CRLM). All under the premises having sufficient technical possibilities and surgeons experience.

In conclusion, with this case we have shown the feasibility of the ALPPS procedure in a newly faced tumor-entity. Furthermore, the importance of an intraoperative histological evaluation of the parenchyma to estimate the individual risk for liver failure can be shown in this case. Also, deciding for ALPPS intraoperatively shows to be feasible.

## Funding

Nothing to declare.

## Ethical approval

No ethical approval for Case Report nessecary. Written informed consent from the patient was obtained.

## Consent

Written informed consent was obtained from the patient for publication of this case report and accompanying images. A copy of the written consent is available for review by the Editor-in-Chief of this journal on request.

## Author contribution

Dmitri Schepelew - writing the paper.

Tim Reese - writing the paper.

Katja Horling - writing the paper, pathological work-up.

Christian Frenzel – patient work up, final approval of manuscript.

York von Rittberg – critical review of the manuscript, final approval of manuscript.

Georgios Makridis – critical review of the manuscript, final approval of manuscript.

Mohammad-H. Fard-Aghaie – critical review of the manuscript, final approval of manuscript.

Kim Caroline Wagner – critical review of the manuscript, final approval of manuscript.

Alexandros Kantas – critical review of the manuscript, final approval of manuscript.

Karl J. Oldhafer – writing the paper, critical review of the manuscript, final approval of manuscript.

## Registration of research studies

N/A.

## Guarantor

Tim Reese and Karl J. Oldhafer.

## Provenance and peer review

Not commissioned, externally peer-reviewed.

## Declaration of Competing Interest

Nothing to declare.

## References

[1] Pachera S., Nishio H., Takahashi Y., Yokoyama Y., Oda K., Ebata T., Igami T., Nagino M. (2008). Undifferentiated embryonal sarcoma of the liver: case report and literature survey. J. Hepatobiliary. Surg..

[2] Faraj W., Mukherji D., El Majzoub N., Shamseddine A., Shamseddine A., Khalife M. (2010). Primary undifferentiated embryonal sarcoma of the liver mistaken for hydatid disease. World J. Surg. Oncol..

[3] Yedibela S., Reck Th., Ott R., Müller V., Papadopoulos Th., Hohenberger W. (2000). Undifferenziertes, embryonales Sarkom als seltene Ursache einer Leberruptur beim Erwachsenen. Chir..

[4] Hong W.J., Kang Y.N., Kang K.J. (2014). Undifferentiated embryonal sarcoma in adult liver. Korean J. Pathol..

[5] Weitz J., Klimstra D.S., Cymes K., Jarnagin W.R., D’Angelica M., La Quaglia M.P., Fong Y., Brennan M.F., Blumgart L.H., DeMatteo R.P. (2007). Management of primary liver sarcomas. Cancer.

[6] Schadde E., Schnitzbauer A.A., Tschuor C., Raptis D.A., Bechstein W.O., Clavien P.-A. (2015). Systematic review and meta-analysis of feasibility, safety, and efficacy of a novel procedure: associating liver partition and portal vein ligation for staged hepatectomy. Ann. Surg. Oncol..

[7] Oldhafer K.J., Donati M., Maghsoudi T., Ojdanić D., Stavrou G.A. (2012). Integration of 3D volumetry, portal vein transection and in situ split procedure: a new surgical strategy for inoperable liver metastasis. J. Gastrointest. Surg..

[8] Schnitzbauer A.A., Lang S.A., Goessmann H., Nadalin S., Baumgart J., Farkas S.A., Fichtner-Feigl S., Lorf T., Goralcyk A., Hörbelt R. (2012). Right portal vein ligation combined with in situ splitting induces rapid left lateral liver lobe hypertrophy enabling 2-Staged extended right hepatic resection in small-for-size settings. Ann. Surg..

[9] Agha R.A., Borrelli M.R., Farwana R., Koshy K., Fowler A., Orgill D.P., For the SCARE Group. The SCARE (2018). Statement: updating consensus surgical CAse REport (SCARE) guidelines. Int. J. Surg..

[10] Bhatt N., Deady S., Gillis A., Bertuzzi A., Fabre A., Heffernan E., Gillham C., O’Toole G., Ridgway P.F. (2016). Epidemiological study of soft-tissue sarcomas in Ireland. Cancer Med..

[11] Hong J.C., Kim J., Browning M., Wagner A., Lerret S., Segura A.D., Zimmerman M.A. (2017). Modified associating liver partition and portal vein ligation for staged hepatectomy for hepatoblastoma in a small infant: how far can we push the envelope?. Ann. Surg..

[12] Qu C., Qu L., Zhu C., Wang Z., Cao J. (2018). Treatment of primary hepatic neuroendocrine tumors with associating liver partition and portal vein ligation for staged hepatectomy (ALPPS): a case report and literature review. Medicine (Baltimore).

[13] Akbulut S., Cicek E., Kolu M., Sahin T.T., Yilmaz S. (2018). Associating liver partition and portal vein ligation for staged hepatectomy for extensive alveolar echinococcosis: first case report in the literature. World J. Gastrointest. Surg..

[14] Linecker M., Stavrou G.A., Oldhafer K.J., Jenner R.M., Seifert B., Lurje G., Bednarsch J., Neumann U., Capobianco I., Nadalin S. (2016). The ALPPS risk score: avoiding futile use of ALPPS. Ann. Surg..

[15] Oldhafer K.J., Stavrou G.A., van Gulik T.M. (2016). ALPPS—where do we stand, where do we go? Eight recommendations from the first international expert meeting. Ann. Surg..

[16] Stavrou G.A., Donati M., Fard-Aghaie M.H., Zeile M., Huber T.M., Stang A., Oldhafer K.J. (2017). Did the international ALPPS meeting 2015 have an impact on daily practice? The Hamburg Barmbek experience of 58 cases. Visc. Med..

